# Climate Trends at a Hotspot of Chronic Kidney Disease of Unknown Causes in Nicaragua, 1973–2014

**DOI:** 10.3390/ijerph18105418

**Published:** 2021-05-19

**Authors:** Zoe E. Petropoulos, Oriana Ramirez-Rubio, Madeleine K. Scammell, Rebecca L. Laws, Damaris Lopez-Pilarte, Juan José Amador, Joan Ballester, Cristina O’Callaghan-Gordo, Daniel R. Brooks

**Affiliations:** 1 Department of Environmental Health, Boston University School of Public Health, Boston, MA 02118, USA; mls@bu.edu (M.K.S.); rebecca.laws@gmail.com (R.L.L.); 2 ISGlobal, 08003 Barcelona, Spain; oriana.ramirez@isglobal.org (O.R.-R.); joan.ballester@isglobal.org (J.B.); cristina.ocallaghan@isglobal.org (C.O.-G.); 3 Department of Epidemiology, Boston University School of Public Health, Boston, MA 02118, USA; dalp1342@gmail.com (D.L.-P.); juanjoseamador3011@gmail.com (J.J.A.); danbrook@bu.edu (D.R.B.); 4 Universitat Pompeu Fabra (UPF), 08002 Barcelona, Spain; 5 Faculty of Health Sciences, Universitat Oberta de Catalunya, 08018 Barcelona, Spain; 6 CIBER Epidemiología y Salud Pública (CIBERESP), 28029 Madrid, Spain

**Keywords:** heat stress, occupational heat exposure, historical weather trends, Central America

## Abstract

An ongoing epidemic of chronic kidney disease of uncertain etiology (CKDu) afflicts large parts of Central America and is hypothesized to be linked to heat stress at work. Mortality rates from CKDu appear to have increased dramatically since the 1970s. To explore this relationship, we assessed trends in maximum and minimum temperatures during harvest months between 1973 and 2014 as well as in the number of days during the harvest season for which the maximum temperature surpassed 35 °C. Data were collected at a weather station at a Nicaraguan sugar company where large numbers of workers have been affected by CKDu. Monthly averages of the daily maximum temperatures between 1996 and 2014 were also compared to concurrent weather data from eight Automated Surface Observing System Network weather stations across Nicaragua. Our objectives were to assess changes in temperature across harvest seasons, estimate the number of days that workers were at risk of heat-related illness and compare daily maximum temperatures across various sites in Nicaragua. The monthly average daily maximum temperature during the harvest season increased by 0.7 °C per decade between 1973 and 1990. The number of days per harvest season with a maximum temperature over 35 °C increased by approximately five days per year between 1974 and 1990, from 32 days to 114 days. Between 1991 and 2013, the number of harvest days with a maximum temperature over 35 °C decreased by two days per year, and the monthly average daily maximum temperature decreased by 0.3 °C per decade. Comparisons with weather stations across Nicaragua demonstrate that this company is located in one of the consistently hottest regions of the country.

## 1. Introduction

Over the past several decades in Central America, clinicians, researchers and public health agencies have identified an epidemic of chronic kidney disease of uncertain etiology (CKDu), also termed Mesoamerican Nephropathy (MeN) [1,2]. CKD mortality rates in El Salvador and Nicaragua are among the highest in the world [3], with these deaths disproportionally occurring in certain regions of these and other Central American countries, mostly in the Pacific lowlands [4,5].

Community prevalence studies, mostly conducted in El Salvador and Nicaragua, indicate a prevalence of around 13–18% and a concentration of disease among males, workers in manual occupations, such as sugarcane and other agriculture, brick manufacture, mining and fishing, and individuals substantially younger than typical CKD patients in the United States [6,7,8,9,10,11,12,13,14]. Some research suggests that persons living and working at low altitudes are at greater risk of CKD than those living and working in the same occupations at higher altitudes, where temperatures are cooler [4,7,8,11,12].

Many researchers have hypothesized that heat exposure combined with strenuous work and dehydration is a likely factor in a multifactorial causal web [7,12,15,16,17,18,19,20,21]. Several studies conducted at sugar companies in Costa Rica, El Salvador and Nicaragua have found that workers are exposed to very high wet-bulb globe temperatures (WBGT) while working in extremely strenuous jobs [22,23,24,25,26,27]. Workers performing the most strenuous tasks in the hottest environments had higher risk of kidney injury and greater declines in kidney function over a harvest season when compared to workers conducting less strenuous tasks [28,29,30,31].

Historic evidence indicates a notable increase of CKDu cases, including an almost nine-fold increase in the Guanacaste region of Costa Rica, beginning in the 1970s [32,33]. Mortality data from Nicaragua are available from the 1990s and show substantial increases particularly in the northwestern region of Nicaragua, which is the location of the largest sugar-producing company in Nicaragua—the Ingenio San Antonio (ISA) [34,35]. Therefore, examining whether ambient temperature has increased in this region over the past several decades could inform the heat stress hypothesis.

Two papers have evaluated climatic trends in Central America as a whole in the latter part of the 20th century. The first, using precipitation data from 105 weather stations and temperature data from 48 weather stations across Central America and northern South America for the years 1961–2003, described a significant increase in the percentage of warm days and warm nights (2.5% and 1.7% per decade, respectively) [36]. It also documented that daily maximum and minimum temperatures increased during this time period by 0.3 °C and 0.2 °C per decade. The second paper, using a combination of monitored and modeled data for 1970–1999, described a significant increase in annual average temperatures, particularly in Guatemala, El Salvador, southern Mexico, Costa Rica and the Caribbean coast of Nicaragua [37].

While these papers examined long-term climatic trends in Central America at a relatively large spatial scale and other studies have examined snapshots of ambient conditions at workplaces in this region, little research has been conducted on long-term climatic changes at the micro-environments relevant to workers at risk of CKDu. The aims of this study were to (1) describe the weather conditions over the past four decades and assess changes in temperature over time, particularly during the sugarcane harvest months (between December and May), at Ingenio San Antonio; (2) estimate the number of days per year that harvest workers at ISA are at risk of heat-related illness; and (3) compare temperatures at ISA to other weather stations across Nicaragua.

## 2. Methods

### 2.1. Data Sources

Weather data were collected at the main facility of the ISA, located in the town of Chichigalpa, Nicaragua (12°31′52.0″ N, 87°03′04.6″ W). Chichigalpa is near the Pacific coast in northwestern Nicaragua in the Department of Chinandega, which is considered to be one of the central foci of this epidemic. Since 1973, a weather station at ISA has measured atmospheric conditions using an aspirated psychrometer, thermohygrograph, evaporimeter, anemometer, anemograph, pluviograph, pluviometer and heliograph. Daily atmospheric data include the minimum, mean and maximum temperature (°C); minimum, mean and maximum relative humidity (%); hours of sunlight per day; wind speed (km/24 h); evaporation (mm) and precipitation (mm). According to the company report, relative humidity data were unreliable prior to 2000 because of the proximity to a cooling stack.

A trained agronomist engineer retrieved data from the weather station every morning and performed a comparison to data from a digital weather station (Watch Dog 2900ET), also located at ISA, as well as to summary data from the previous three years. The weather station instrumentation was calibrated and maintained monthly, or more often if needed. In these analyses, we used daily weather data collected from May 1973 to August 2014.

Historical weather data from Automated Surface Observing System (ASOS) Network weather stations in Nicaragua (located in Bluefields, Puerto Cabezas, Managua, Rivas, Juigalpa, Jinotega, and Chinandega) were downloaded from Weather Underground (www.wunderground.com, accessed on 26 March 2018) for 1996–2014 using the rwunderground package in R. Hourly data on temperature (°C) were available for only a few—primarily daytime—hours each day [38].

### 2.2. Statistical Analysis

Analyses were performed in SAS 9.4 (SAS Institute, Cary, NC, USA) and graphics were produced in R (R Core Team, 2014).

#### 2.2.1. Summary of Weather Conditions at ISA, 1973–2014

We summarized the daily minimum, maximum and mean temperature at ISA between 1973 and 2014. The daily minimum, maximum and mean relative humidity at ISA were summarized for the years 2000–2014, as older data were deemed unreliable. Extreme values were evaluated as potential outliers by calculating the number of standard deviations (SD) away from the mean.

#### 2.2.2. Changes in Average Temperatures and Temperature Extremes

We calculated the monthly averages of daily maximum temperatures for the months during sugarcane harvest (December–May) for all years. We used simple linear regression and Mann–Kendall tests to determine whether monthly averages increased over time. After observing an apparent change in slope approximately midway through the period, we employed automated piecewise regression to assess which trend lines best fit the data using Joinpoint software Version 4.6.0.0 (National Cancer Institute, Calverton, MD, USA). Two breakpoints were identified: April 1990 and November 2013. Because the latter date was 6 months before the end of the weather data, we ignored the second breakpoint and considered the slopes for May 1973–April 1990 and April 1990–November 2013.

We also calculated the monthly averages of daily mean temperatures. The monthly maximum and the monthly minimum temperature were identified as the highest and lowest temperatures recorded each month, respectively. We ran simple linear regressions and Mann–Kendall tests for trends on these data as well.

We calculated the number of days during the harvest season for which the measured maximum temperature exceeded 35 °C and fit a simple linear regression model and a piecewise model using the year as the predictor to determine whether the number of days over 35 °C increased over time. We applied the same breakpoint as identified in the model predicting monthly average daily maximum temperature.

#### 2.2.3. Heat Index, 2000–2014

The heat index combines relative humidity with air temperature in a composite index value and is useful for assessing the risk of heat-related illnesses in occupational settings, particularly when work is taking place indoors or in the shade. Lacking hourly data at ISA, we were not able to calculate a daily maximum HI value for each day because we could not ensure that maximum values of humidity and temperature occurred at the same time. In many instances, the use of both daily maximum humidity and daily maximum temperature to calculate HI produced unrealistic or impossible values (e.g., 45% of calculated values were >60 °C); thus, we instead used maximum temperature with mean humidity, mean temperature with maximum humidity and mean temperature with mean humidity. It should be emphasized that these HI values do not accurately reflect the maximum HI on a given day, so our comparisons to U.S. Occupational Safety and Health Administration (OSHA) guidelines could under or over-estimate the overall risk to workers, depending on the extent of misclassification. HI analyses were restricted to 2000–2014, the period during which humidity data were considered reliable. Regression models were not fit for the data using HI because of the unreliability of the humidity data before 2000. However, we report a summary of the mean HI values for 2000–2014 and compare them with OSHA guidance [39].

#### 2.2.4. Comparison to Other Nicaraguan Weather Stations

We plotted the monthly average maximum temperature at all eight weather stations in Nicaragua to compare historical weather trends at the different sites across the country between 1996 and 2014. We used the weathermetrics package in R to convert °F to °C for the datasets downloaded from Weather Underground, as well as for the calculations of HI using the U.S. National Weather Service’s algorithm [40,41].

## 3. Results

### 3.1. Summary of Weather Conditions at ISA, 1973–2014

The average daily maximum temperature during the time period of interest was 33.6 °C (SD: 2.2, range: 21.0–40.8), while the average daily minimum temperature was 21.9 °C (SD: 2.0, range: 12.2–29.0) (Table 1). The average daily maximum temperature was higher for harvest months than non-harvest months (mean: 34.7 °C vs 32.5 °C), while precipitation was higher, on average, in non-harvest months than in harvest months (mean: 7.6 mm vs 2.5 mm). These findings are congruous with the two seasons that are widely recognized in this region: a hotter, drier harvest season and a slightly cooler, wetter non-harvest rainy season. The average daily maximum relative humidity was 94.6% for all months and was slightly lower for harvest compared to non-harvest months (92.3% vs. 96.8%; see Table 1). The average daily mean relative humidity was higher during non-harvest months than harvest months (73.4% vs. 61.3%).

### 3.2. Temperature and Heat Index Trends at ISA

#### 3.2.1. Monthly Average Temperatures and Monthly Extreme Temperatures

**Figure 1:**
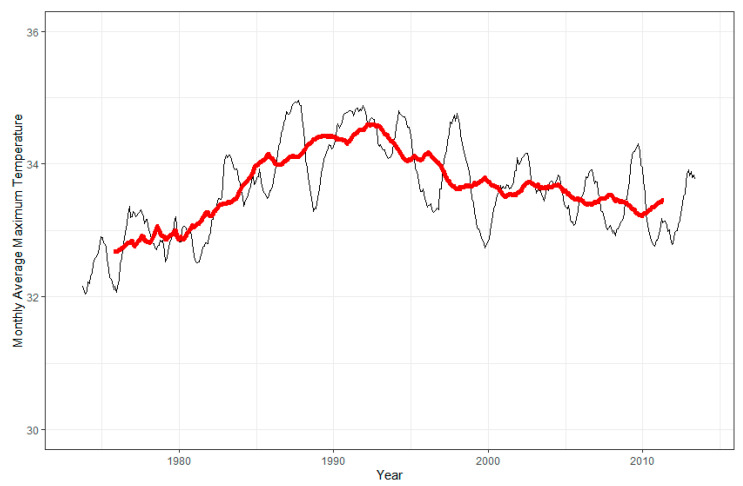
One-year (black line) and five-year (red line) rolling averages of monthly-averaged daily maximum temperatures at ISA, all months in the period May 1973–November 2013.

Between May 1973 and April 1990, the monthly average daily maximum temperature increased by 0.07 °C per year (standard error (SE): 0.002; *p*-value < 0.0001), and it decreased by 0.03 °C per year (SE: 0.001; *p*-value < 0.0001) between April 1990 and November 2013. Figure 1 presents the one-year (black line) and five-year (red line) rolling averages of monthly-averaged daily maximum temperatures at ISA for all months between May 1973–November 2013. These correspond to a 0.7 °C per decade increase between 1973 and 1990 and 0.3 °C per decade decrease between 1990 and 2013, respectively. The Mann–Kendall tests for trends (not shown) demonstrated a positive trend (tau: 0.07, *p*-value: 0.01).

Monthly minimum temperature was inversely associated with time (−0.004 °C/month; *p*-value: 0.03). The monthly average daily mean temperature did not change meaningfully over time. The monthly maximum temperature increased 0.004 °C per month (*p*-value: 0.01), corresponding to a 0.024 °C increase per harvest season. The Mann–Kendall tests for trends for monthly minimum and monthly maximum temperatures demonstrated a negative (tau: −0.08, *p*-value: 0.01) and positive trend (tau: 0.1, *p*-value: 0.001), respectively.

#### 3.2.2. Days per Harvest Season above 35 °C

**Figure 2:**
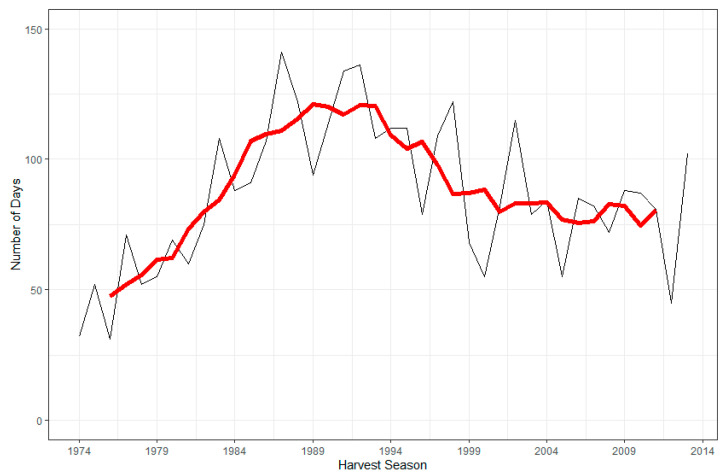
Number of days during harvest months (December–May) with a maximum temperature over 35 °C, 1974–2013, with 5-year rolling average (red line).

Figure 2 depicts the number of days per harvest season (December through May) with a daily maximum temperature over 35 °C during the period from the 1974–1975 harvest season to the 2013–2014 harvest season. To account for the non-linearity of the data, we applied an equivalent breakpoint (1989–1990 harvest season) to the one identified previously with the monthly average daily maximum temperature data. Between 1974 and 1990, the number of days during the harvest with a maximum temperature over 35 °C increased by 5.1 per year (Table 2) (standard error: 0.74; *p*-value < 0.0001), while the number decreased between 1991 and 2014 by 2.2 per year (Table 2) (SE: 0.46; *p*-value < 0.0001). Between 1974 and 1990, the number of days per harvest season with a maximum temperature over 35 °C increased from 32 days to 114 days. This corresponds to an increase from 17.6% of the harvest season with a maximum temperature over 35 °C in 1974 to 62.6% in 1990. Between 1991 and 2013, an average of 50.0% of harvest days had a maximum temperature above 35 °C.

#### 3.2.3. Heat Index

**Figure 3:**
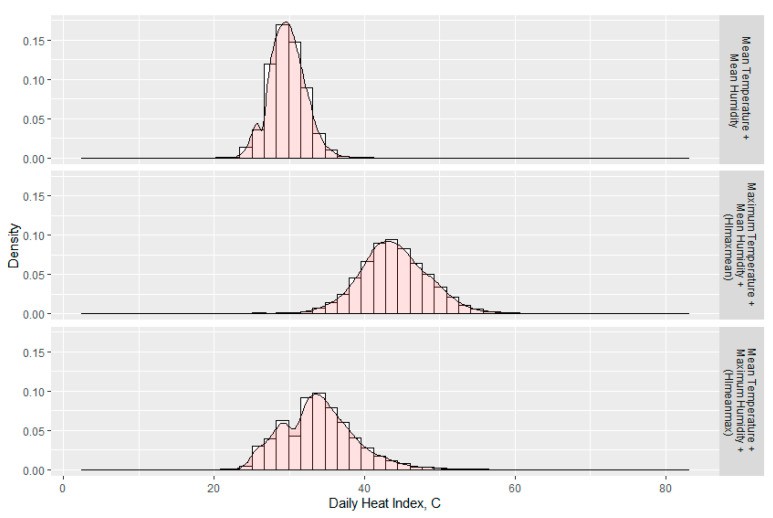
Distributions of all daily HI summaries calculated using three combinations of mean and maximum temperature and humidity, 2000–2014.

Figure 3 shows the distributions of HI calculated using the three combinations of mean and maximum temperature and humidity. As expected, the combination of mean humidity and mean temperature produced a narrower distribution of relatively cooler HI estimates, while maximum temperature and mean humidity (HI_maxmean_) and mean temperature and maximum humidity (HI_meanmax_) produced more normal-looking distributions with higher means. For the purpose of estimating daily maximum HI, we used both of these latter approaches.

The percentage of days during the harvest season that exceeded OSHA’s low heat index category was 99% for HI_maxmean_ and approximately 60% for HI_meanmax_ (Table 3). HI_maxmean_ reached OSHA’s highest category for HI-based risk levels in more than 35% of harvest days, while only about 3% of harvest days reached this level using HI_meanmax_.

### 3.3. Comparison to Other Nicaraguan Weather Stations

**Figure 4:**
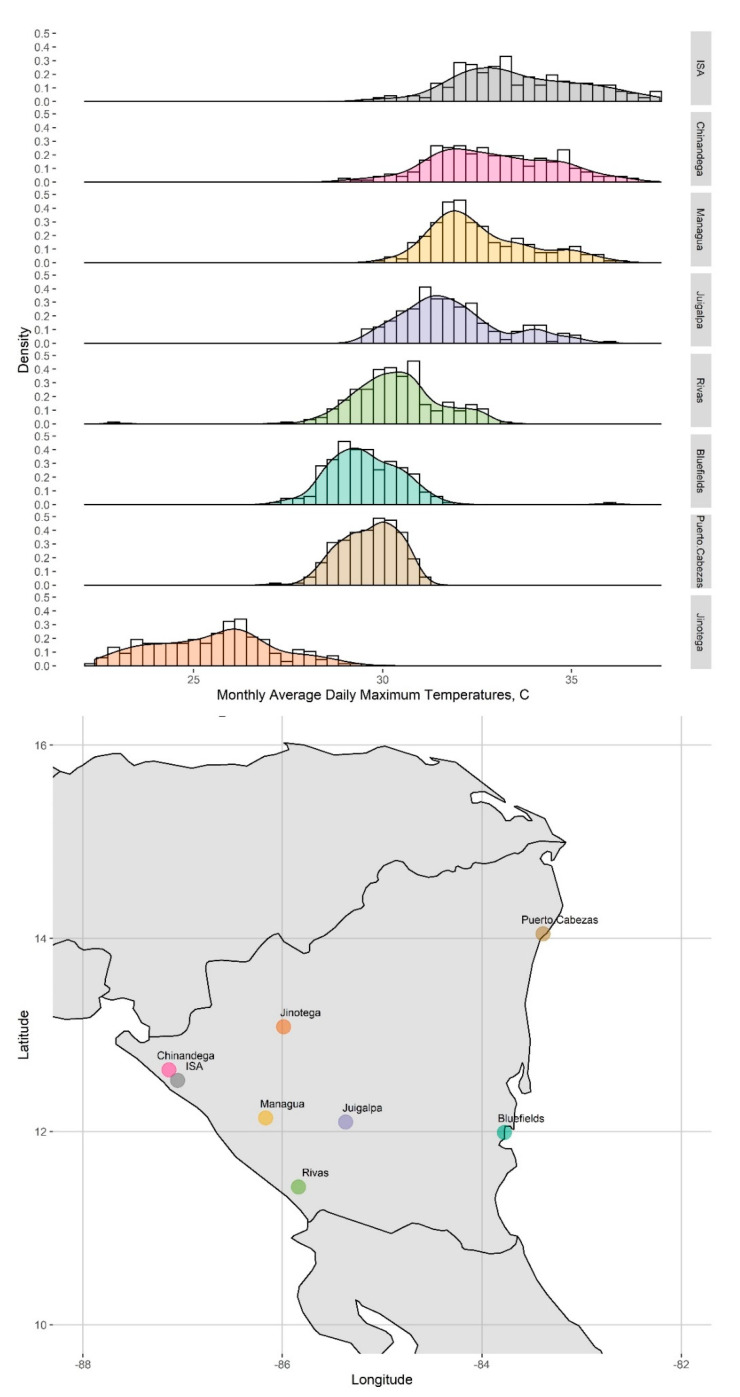
Histograms of monthly-averaged daily maximum temperatures at all seven ASOS Network monitors and ISA, 1996–2014, and map of the locations of each monitor.

Figure 4 shows the distribution of monthly-averaged daily maximum temperature at all eight sites (ISA in gray). ISA and nearby Chinandega have overall higher monthly average maximum temperatures compared to the other sites, with Managua and Juigalpa closely following. Chinandega and Managua, both near the Pacific coast in western Nicaragua, are the two closest sites to ISA geographically.

## 4. Discussion

In this study, we found that the average daily maximum temperature at ISA, especially during the harvesting months, was higher than in other parts of Nicaragua, and that it increased during the period between 1973 and 1990. An earlier study of climatic trends in Central America found that western Nicaragua, where ISA is located, was hotter than the rest of the country during the period between 1970–1999 [37]. We confirmed this trend through 2014 based on a comparison to other weather monitoring stations across Nicaragua. This region of Nicaragua also has the highest CKD mortality rates [34].

We also estimated that workers at this sugarcane facility were at a high or very high risk of heat-related illness on 20–88% (depending on the heat index estimates used) of the working days during the harvesting seasons between 2000 and 2014 (based on OSHA guidance), which might have had adverse health effects on these workers, including renal damage [31,42,43]. OSHA provides guidelines for employers regarding workplace heat hazards based on the heat index. Even at a heat index between 32.8 °C to 39.4 °C (corresponding to the “moderate” category in Table 3), when workers are wearing protective clothing, working in full sunshine or performing strenuous activities—characteristics that apply to multiple job categories at ISA—OSHA recommends that employers modify work schedules and conduct physiological and verbal monitoring for heat-related illness among workers (in addition to more basic precautions, such as training workers, providing adequate water and acclimatizing workers). In the years toward the end of the period covered by this analysis, ISA instituted a multi-component program to reduce heat stress among workers in the highest-risk jobs, which has continued and been expanded to the present time [44].

Evidence suggests that mortality due to CKD has increased in Central America since the 1970s, with estimates of a nine-fold increase in the Guanacaste region of Costa Rica from 1970–2012, an approximately 1.5-fold increase in El Salvador from 2000–2009 and a 2.5-fold and 5-fold increase in the Nicaraguan departments of Chinandega and León, respectively, from 1992–2005 [3,32,33,34]. We were particularly interested in whether there was a change in temperature during this time period consistent with the increase in CKDu mortality trends. The results of our study were neither wholly consistent nor inconsistent with this hypothesis. The monthly average daily maximum temperature during harvest months increased overall by 1.1 °C between 1974 and 1990. The percentage of days during the harvest season during which the maximum temperature exceeded 35 °C also markedly increased during this period, rising from 17.6% of harvest season days in 1974 to 62.6% in 1990. These findings are consistent with previous research by Aguilar and colleagues, which found that the annual maximum daily maximum temperature increased by 0.3 °C per decade between 1961–2003 [36]. On the other hand, Hidalgo et al. found that while annual average temperatures increased in Guatemala, El Salvador, Costa Rica and the Caribbean coast of Nicaragua between 1970–1999, the increase in annual average temperature in western Nicaragua was much smaller or non-existent [37]. Between 2000–2013, we observed a decadal decrease of 0.3 °C in monthly average daily maximum temperature during harvest months and an annual decrease of 2.2 days per harvest season that had a maximum temperature over 35 °C. This period between 2000 and 2013 overlaps with the purported “global warming hiatus” during which the rate of increase of global surface temperatures temporarily slowed down. This trend in the ISA data is also reflected in other climate analyses from the region [45,46]. However, average temperatures in years since 2013 have largely been the hottest recorded in this region, mirroring global climate trends. Our analyses unfortunately do not include data from after 2014, including 2015 and 2016, during which a major El Niño–Southern Oscillation (ENSO) episode catalyzed a string of record-breaking years. This could explain the second breakpoint we found in our analyses in November 2013. The ENSO may also explain the patterns observed in Figure 1 and Figure 2. There were multiple El Niño events in the late 1980s and early-to-mid 1990s, which may have contributed to the warming temperatures seen in those years. Our data are also consistent with potential cooling from La Niña episodes in the 1970s, late 1990s and mid-2000s. However, the extent of these impacts cannot be inferred from our data alone.

The ability to interpret the relationship between the observed changes in temperature and CKDu incidence in Nicaragua is limited by several factors, including (i) the lack of data on incidence of disease, which necessitates the use of overall CKD mortality as a proxy measure, and the absence of data on even this proxy measure during the 1970s and 1980s; (ii) a lack of understanding on how heat stress might lead to disease (e.g., short-term acute vs. long-term chronic exposure, and the extent to which the impact of heat stress might be a cumulative linear association vs. a threshold phenomenon) and (iii) very limited knowledge about the natural history of disease—specifically, the average number of years between disease incidence and mortality.

Our analyses are subject to additional limitations. While temperature metrics that capture humidity (such as HI or WBGT) are particularly important when studying the effects of temperature on the health of outdoor workers, we had reliable humidity data only after 1999 and were therefore unable to assess longer-term trends in heat indexes. Additionally, the ISA temperature and humidity data were reported as daily minimums, maximums and means, so we were unable to determine the maximum heat index on a given day and likely have inaccurate estimates of daily maximum heat indexes. We were unable to conduct an integrated evaluation of temperatures across the workday, also due to a lack of hourly data. Additional data on wind speed and radiation would have also allowed us to estimate WBGT, but these were not available for ISA. Finally, data collected from the ASOS Network stations were only reported for certain hours of the day, so averaging those to daily average conditions is likely to introduce some inaccuracies. We did not estimate daily or monthly minimum values, as we often did not have nighttime data from these monitors. Data from these ASOS Network stations were also only available beginning in 1996, limiting the temporal extent of our comparisons.

If heat stress does play a key role in the CKDu epidemic in Mesoamerica as hypothesized, the at-risk population will continue to grow, with predicted increases in both mean ambient temperature and weather extremes (i.e., heat waves) due to climate change [47,48,49]. The Intergovernmental Panel on Climate Change (IPCC) projects global warming of 1–2 °C by 2065 and 1-3.7 °C by 2100 (relative to 1986–2005 temperatures) under different emissions scenarios [50]. Furthermore, climate models have shown that tropical, low-latitude regions display the earliest significant warming and are also the most vulnerable due to low adaptive capacity [49,51,52]. The Lancet Commission on Health and Climate Change [53] estimates that exposure to more frequent and intense heatwaves is increasing, with an estimated 125 million additional vulnerable adults exposed to heatwaves between 2000 and 2016. The report also describes how increasing ambient temperatures have resulted in an estimated reduction of 5.3% in outdoor manual labor productivity worldwide. These effects are most notable in some of the most vulnerable countries in the world (e.g., at least 10 to 30% decline in labor productivity along the Pacific Coast of Nicaragua).

Understanding the role of heat stress in CKDu etiology is therefore an important component of designing tailored prevention and adaptation strategies aimed at reducing the incidence of this disease in this region. Researchers are already testing the effectiveness of workplace interventions (such as increased hydration practices, more accessible shade and scheduled rest periods, to name a few) aimed at reducing occupational heat stress and the progression or onset of kidney disease [54,55]. Regardless of whether heat stress is implicated in the etiology of CKDu, employers in this region of the world need to be prepared to protect workers against heat-related injuries and illness, as climate change will likely make these hot, humid environments even more dangerous.

## 5. Conclusions

We found that the average daily maximum temperatures at a sugar company with high rates of CKDu in northwestern Nicaragua were higher than in other parts of Nicaragua and that temperatures increased during the period between 1973 and 1990. On a majority of work days during the harvest season, workers were at a moderate or high risk of heat-related illness based on OSHA’s heat index-based risk guidelines. While we cannot draw any causal conclusions from this research, we believe these findings add important context to ongoing research on the role of heat stress in the development of CKDu. The incidence of this disease increased substantially beginning in the 1970s; thus, understanding concurrent changes to environmental exposures during this time, even at an ecological level, is useful for eventually elucidating a cause. Finally, regardless of the connection between chronic heat stress and CKDu, we believe these findings have broader implications for worker safety and health in this region. Temperatures frequently exceed thresholds above which OSHA recommends workplace interventions to avoid heat-related illness.

## Figures and Tables

**Table 1:** Summary statistics for daily temperature, precipitation and relative humidity at ISA, 1973–2014 and 2000–2014.

	May 1973—August 2014 (*n* = 15,037)	Harvest Months Only ^a^, 1973–2014 (*n* = 7503)	Non-Harvest Months Only ^b^, 1973–2014 (*n* = 7534)
	Average (SD), range	Average (SD), range	Average (SD), range
Maximum temperature (°C)	33.6 (2.2), 21–40.8	34.7 (2.0), 24.2–40.8	32.5 (1.8), 21–38.8
Minimum temperature (°C)	21.9 (2.0), 12.2–29.0	21.2 (2.3), 12.7–29.0	22.5 (1.3), 12.2–29.0
Mean temperature (°C)	27.7 (1.4), 20.7–36.0	28.0 (1.7), 21.0–36.0	27.5 (1.1), 20.7–31.4
Precipitation (mm)	5.1 (15.2), 0.0–584	2.5 (10.2), 0.0–285	7.6 (20.3), 0.0–584
	January 2000–August 2014 (*n* = 5357)	Harvest Months Only, 2000–2014 (*n* = 2703)	Non-Harvest Months Only, 2000–2014 (*n* = 2654)
	Average (SD), range	Average (SD), range	Average (SD), range
Maximum relative humidity (%)	94.6 (8.0), 23.0–100	92.3 (9.7), 23.0–100	96.8 (4.8), 38.0–100
Minimum relative humidity (%)	40.3 (18.0), 6.0–100	30.2 (12.8), 7.0–100	50.0 (16.9), 6.0–100
Mean relative humidity (%)	67.4 (11.1), 22.0–100	61.3 (8.9), 22.0–100	73.4 (9.6), 37.0–100

^a^ December, January, February, March, April, and May. ^b^ June, July, August, September, October, and November.

**Table 2:** Change in the number of days during harvest with maximum temperature over 35 °C per year between 1974–1990 and 1991–2014.

Time Period	Trend (Standard Error)	*p*-Value
Overall period: 1974–2014	0.42 (0.37)	0.26
Period between 1974–1990 (before breakpoint)	5.10 (0.74)	<0.0001
Period between 1991–2014 (after breakpoint)	−2.16 (0.46)	<0.0001

**Table 3:** Percentage of days meeting criteria for U.S. Occupational Safety and Health Administration heat index-based risk levels at ISA, 2000–2014.

	Maximum Temperature + Mean Humidity		Mean Temperature + Maximum Humidity	
Maximum Risk Level Experienced (OSHA Heat Index Ranges) ^a^	All Months, 2000–2014	Harvest Months Only ^b^, 2000–2014	All Months, 2000–2014	Harvest Months Only ^b^, 2000–2014
**Low** (<32.7 °C)	0.8%	0.4%	40.3%	42.8%
**Moderate** (32.7 °C–39.4 °C)	13.7%	11.2%	47.5%	37.6%
**High** (39.4 °C–46.1 °C)	55.0%	52.2%	10.8%	16.9%
**Very High** (>46.1 °C)	30.6%	36.2%	1.4%	2.7%

^a^ These ranges represent heat index values, incorporating both temperature and relative humidity in a single value. ^b^ December, January, February, March, April, and May.

## Data Availability

Historical weather data from ASOS Network stations are publicly available at www.wunderground.com.
